# Influence of boron supplementation on performance, immunity and antioxidant status of lambs fed diets with or without adequate level of calcium

**DOI:** 10.1371/journal.pone.0187203

**Published:** 2017-11-15

**Authors:** T. Vijay Bhasker, N. K. S. Gowda, D. T. Pal, S. Karthik Bhat, P. Krishnamoorthy, S. Mondal, A. K. Pattanaik, A. K. Verma

**Affiliations:** 1 Animal Nutrition Division, ICAR-Indian Veterinary Research Institute, Izatnagar, India; 2 Animal Nutrition Division, ICAR-National Institute of Animal Nutrition and Physiology, Bangalore, India; 3 ICAR-National Institute of Veterinary Epidemiology and Disease Informatics, Bangalore, India; 4 Animal Physiology Division, ICAR-National Institute of Animal Nutrition and Physiology, Bangalore, India; Leibniz-Institut fur Pflanzengenetik und Kulturpflanzenforschung Gatersleben, GERMANY

## Abstract

Little is known about biological significance of effects of dietary Boron (B) and Calcium (Ca) interaction on health and production of farm animals. This is a preliminary investigation to evaluate the effects of B supplementation in lambs fed diets with (normal) or without adequate (low) levels of Ca. Twenty-four crossbred ram lambs were randomly distributed into four groups with six animals each in a 2x2 factorial design namely, normal-Ca diet (NCa) and low-Ca diet (LCa) fed without or with 40 ppm B (NCaB-40 and LCaB-40). The lambs were fed paddy straw and hybrid napier hay-based total mixed ration (60 roughage: 40 concentrate) during 180 days experimental period. Compared to control, the LCa diet lowered (P<0.01) average daily gain of lambs, but B-supplementation (LCaB-40) of the same nullified the effect. The lowered (P<0.05) total antioxidant activity and humoral immune response in lambs fed LCa diet were restored (P>0.05) to become at par with the control (NCa) upon supplementation of B (LCaB-40). The mRNA expression of SOD1 was lowered (P<0.05) due to LCa diet feeding which too was normalized on B-supplementation to become at par (P>0.05) with the control (NCa). Further, B-supplementation restored lowered (P<0.05) SOD1 gene expression on LCa diet, but enhanced (P<0.05) that in NCaB-40 group, when compared to the control (NCa) diet fed animals. However, these variations were not reflected in the SOD activity in the erythrocytes. The cell-mediated immune response was higher (P<0.05) in lambs fed LCa and LCaB-40 groups and there was no significant interaction between the levels of either Ca or B in diets with the period of immune response measurement. B- supplementation of LCa diet ameliorated tissue degenerative changes in liver and kidney. It was concluded that feeding LCa diet to lambs resulted in reduced growth rate, total antioxidant activity, humoral immune response along with degenerative changes in liver and kidney tissues, but B-supplementation of such diet restored most of these changes and ameliorated histopathological alterations.

## Introduction

The significance of dietary Boron (B) in farm animals is not clearly understood. Boron is a critical micronutrient for plants and the average B content in commonly used tropical animal feeds was reported to be 17.7 ppm [1]. Limited studies in farm animals indicate the benefits of dietary B in improving utilisation of bone forming minerals, antioxidant status, immunity and health. Dietary B (30 g/day) as sodium borate was beneficial in sustaining the metabolic status during periparturient period by diversely promoting glucose metabolism and limiting lipolysis intensity [2, 3] and improving serum levels of calcium (Ca), magnesium (Mg) and phosphorus (P) [4] thereby preventing metabolic disorders in dairy cattle.

Previous studies have indicated the putative role of dietary B in improving antioxidant defence mechanism (superoxide dismutase, catalase and glutathione peroxidase enzyme activities) thereby ameliorating oxidative stress [5] and provide hepato-protective effect against hepatotoxicity induced by carbon tetrachloride in rats [6]. Dietary B-supplementation enhanced immunity by increasing serum levels of tumour necrosis factor (TNF-α) and interferon-gamma (IFN-γ) in pigs [7], reduced inflammatory response against phyto-hemagglutinin (PHA-P) in gilts [8] and increased serum levels of TNF-α in steers inoculated with bovine herpes virus type-1 [9].

Our own previous study in rats fed purified diets has shown that supplementing B to low Ca diet improved both cell mediated and humoral immunity response, increased serum antioxidant status and relative mRNA expression levels of SOD iso-enzymes in liver [10]. However, no such studies are reported in farm animals to understand the role of B in relation to Ca level of diet and its effect on visceral organs. Hence, this investigation was planned to evaluate the role of dietary B on immune response, antioxidant status and growth performance in lambs fed diets with or without adequate Ca.

## Materials and methods

This experiment was conducted in the Experimental Livestock Unit of National Institute of Animal Nutrition and Physiology, Bangalore, India. Animal experiment protocol was approved by Committee for the Purpose of Control and Supervision of Experiments in Animals, Ministry of Environment, Forests and Climate Change, Government of India (No. 3/2014).

### Animal distribution, housing and management

Twenty-four crossbred (Bannur x Mandya) ram lambs (5–6 months age) with an average body weight of 17.8 ± 0.33 kg were randomly distributed into four groups, with six animals each in a 2x2 factorial design viz., normal calcium diet (NCa) and low-calcium diet (LCa) fed without or with 40 ppm boron (NCaB-40 and LCaB-40) supplementation. The lambs were maintained under uniform conditions by housing them in a well ventilated barn with individual feeding facility. All lambs were dewormed using ALBOMAR (Albendazole oral suspension; Virbac Animal Health Pvt. Ltd., Mumbai, India) @ 5–10 ml/lamb at the beginning of the experiment and protected against enterotoxaemia and *peste de petites ruminants* (PPR) with vaccines (Institute of Biological Production, Animal Health and Veterinary Biologicals, Bangalore, India). The feeding experiment was conducted for 180 days period.

### Experimental diet

The ingredient composition of four experimental rations is presented in the Table 1. The lambs were fed paddy straw and hybrid napier hay-based total mixed ration (TMR; with a roughage:concentrate ratio of 60:40). Two types of mineral mixtures were formulated with normal (100%) Ca level (mineral mixture I, Ca 17%) and 50% Ca level (mineral mixture II, Ca 8.50%) as presented in the Table 2. The deficit of phosphorus in mineral mixture II was adjusted by adding calculated amount of diammonium phosphate.

**Table 1:** Ingredient and chemical composition of experimental diets.

Ingredient (%)	Dietary groups^†^			
	NCa	LCa	NCaB-40	LCaB-40
Paddy straw	30	30	30	30
Hybrid napier hay	30	30	30	30
Concentrate mixture^¶^	40	40	40	40
Mineral mixture I (17% Ca)	+	–	+	–
Mineral mixture II (8.5% Ca)	–	+	–	+
Boron (ppm)	–	–	40	40
**Proximate Constituents (%)**				
Dry matter	92.60	91.84	89.73	91.51
Organic matter	89.12	89.42	89.75	89.53
Ash	10.88	10.58	10.25	10.47
Crude protein	12.23	13.47	12.55	12.68
Ether extract	1.42	1.47	1.68	1.63
Crude fibre	22.72	20.93	20.27	23.63
Neutral detergent fibre	66.23	65.97	66.21	66.01
Acid detergent fibre	44.28	44.61	44.31	43.02
Nitrogen free extract	52.75	53.55	55.25	52.59
**Analysed macro minerals (%)**				
Calcium	0.53	0.36	0.54	0.36
Magnesium	0.30	0.26	0.27	0.28
Phosphorus	0.34	0.29	0.30	0.32
**Analyzed micro minerals (ppm)**				
Copper	12.52	12.36	12.47	12.96
Zinc	56.2	55.7	58.1	56.8
Manganese	23.6	24.2	23.9	24.0
Boron^¶¶^	8.42	8.76	54.55	51.88

^**†**^Normal calcium diet (NCa) and Low calcium diet (LCa) supplemented with 40 ppm boron (NCaB-40 and LCaB-40) in the form of sodium borate; ppm: Parts per million

^¶^Composed of 39.50% crushed maize grain, 30% wheat bran, 30.25% soybean meal and 0.25% common salt, added with vitamin AD_3_ at 20 g/100 kg.

^¶¶^ The 40 ppm B-supplementation raised basal B level (8.4 ppm) of normal-calcium (NCa) and low calcium diet (LCa) in proportionate amounts ensuring their desired levels in the NCaB-40 and LCaB-40 diets. Appropriate quality control measures during estimation prevented Boron loss.

**Table 2:** Composition of mineral mixtures (100g).

Ingredient (g)	Mineral mixture I	Mineral mixture II
Dicalcium phosphate	50.00	25.00
Calcite	17.00	8.50
Magnesium sulphate	13.00	13.00
Manganese sulphate	1.70	1.70
Zinc sulphate	3.30	3.30
Copper sulphate	0.50	0.50
Cobalt chloride	0.01	0.01
Ferrous sulphate	1.20	1.20
Sodium selenite	0.002	0.002
Diammonium phosphate	-	22.50
Sucrose	-	11.01
Common salt	13.28	13.28

### Feeding regime

Weighed quantities of respective diets were offered to lambs of various groups twice daily at 9.00 AM and 3.00 PM so as to meet their nutrient requirements [11]. The diets NCa & NCaB-40 and LCa & LCaB-40 were additionally supplemented with mineral mixture I and II, respectively, at the rate of 2% (on fresh basis) of diet. Further, diets NCaB-40 and LCaB-40 were supplemented with B at the rate of 40 mg per kg diet in the form of sodium borate (Borax, AR grade). The feed residue was weighed in the next day morning and composited at weekly intervals to estimate dry matter content. Body weight of each animal was recorded at fortnightly intervals and the feeding schedule was adjusted accordingly. Drinking water was made available to each animal all the time.

### Proximate and mineral analysis of experimental diet

The samples of diets offered were dried and ground in a hammer mill to a fineness of 1mm size and analysed for proximate principles [12] and fibre fractions i.e., neutral detergent fibre (NDF) and acid detergent fibre (ADF) [13]. The feed samples were taken in pre-weighed silica crucibles and oven dried at 80°C for 24 h, decarbonized and ashed at 600°C in a muffle furnace for 3 h. The total ash was digested with 5N HCl over a hot plate for 15 min, cooled and filtered through Whatman filter paper (No. 41) into volumetric flasks. Mineral contents in feed samples were estimated using inductively coupled plasma optical emission spectrophotometer (ICP-OES; Optima 800, Perkin Elmer, Shelton, USA) adopting the operating conditions suggested by the manufacturer [10]. Mineral standard for each element was prepared from ICP multi-element standard solution (Merck Millipore) to arrive at a concentration of 1000 mg/L (stock solution) except for phosphorus, which was prepared from potassium dihydrogen phosphate (KH_2_PO_4_). For sample preparation and storage, quartz flasks were used instead of borosilicate to avoid contamination with B.

### Cell mediated immune response

After 150 days of experimental feeding, six sheep from each dietary group were assessed for cell mediated immune (CMI) response using *in vivo* DTH (delayed-type hypersensitivity) test against PHA-P (Phytohemagglutinin-*Phaseolus vulgaris)*. A stock solution of PHA-P was prepared in phosphate buffer saline at a concentration of 160 μg/ml and autoclaved, which was later filtered through 0.2 μm membrane filter before use. It was suitably diluted to provide 20 μg PHA-P per inoculum in a volume of 125 μl (5 units in micro syringe). The skin area to be tested (both sides of neck region) was cleaned and shaved 24 h prior to performing the DTH test. An area of about one square cm was encircled with a marker pen, on both sides of the neck region. The thickness of the skin was measured by using Vernier calipers, which represented the basal (0 h) value. All animals were injected intradermally with 125 μl of PHA-P (20 μg per 125μl) solution on one side and normal saline solution as a negative control on the other side of neck region. The thickness of the skin was subsequently measured at 12, 24, 36 and 48 h post-inocultion.

### Humoral immune response

After 160 days of experimental feeding, six sheep from each dietary group were antigenically challenged with subcutaneous injection of a commercial PPR vaccine. Following 14 days of injection, blood samples were withdrawn from jugular vein of the sheep and collected into dry, sterilized centrifuge tubes and allowed for clotting. After clotting, the tubes were centrifuged to collect sera. The collected sera samples were stored in refrigerated condition (-20°C) till further analysis. Antibody titer against PPR vaccine was determined using competitive enzyme linked immunosorbent assay kit procured from Indian Veterinary Research Institute, Izatnagar, India [14] and expressed as percent of inhibition.

### Superoxide dismutase (SOD) activity

On 180^th^ day of the experimental feeding, blood (2 ml) was collected from jugular vein of all sheep into vials with anticoagulant (acid citrate dextrose; 300 μl/2 ml blood), and centrifuged at 2000 rpm for 15 min at 4°C for separation of plasma and buffy coat. Haemolysate (1:20 dilution) was prepared by mixing 0.5 ml RBC suspension with 4.5 ml of stabilizing solution (EDTA, 2.7 mM and 0.7 mM, 2-mercaptoethanol). The SOD activity in haemolysate was measured using nitro blue tetrazolium as substrate after suitable dilution as per the method of Marklund andMarklund [15] with certain modifications as suggested by Minami and Yoshikawa [16]. One unit of SOD activity was defined as the amount of enzyme which inhibited the auto-oxidation of pyrogallol by 50% under the given experimental condition. The values were expressed as units per mg of haemoglobin.

### Total antioxidant activity (TAA)

On 180^th^ day of experimental feeding, blood (2 ml) from each sheep was collected into cleaned, dry, sterilized centrifuged tubes and allowed for clotting and later centrifuged to collect sera. The TAA in serum samples was determined as per the protocol of total antioxidant capacity colorimetric assay kit (Catalog# K274-100, Bio Vision, USA). One μl serum sample was taken in micro centrifuge tube and the total volume was adjusted to 100 μl by adding 99 μl of distilled water. After proper vortexing, the diluted serum samples were transferred into individual wells. Exactly, 100 μl of Cu^2+^ working solution was added to all the standard (trolox; 6-hydroxy-2,5,7,8-tetramethylchroman-2-carboxylic acid) and sample wells. The plate was covered and incubated at room temperature for 90 min. Absorbance was recorded at 570 nm using the ‘Multiskan GO’ (Thermofischer, Finland). Absorbance at 570 nm as a function of trolox concentration was plotted against the trolox standard curve to determine the sample concentration, expressed as mM trolox equivalent.

### Histopathology

After 180 days of experimental feeding, five sheep from each dietary group were sacrificed by Halal method (jugular vein incision) to collect the vital organs (liver, kidney and spleen) for histopathological investigation. The tissues were collected and immediately fixed in 10% formalin. The sections were stained with haematoxylin and eosin stain [17] and covered with DPX (SRL, India) mounting medium and examined under a light microscope (Nikon eclipse 80i, Japan) to assess the histopathological changes.

### Relative mRNA abundance of SOD1

The liver tissues of sacrificed sheep were collected, washed with Dulbecco’s PBS (Sigma, USA). The samples were then incubatedat 4°C overnight in RNAlater (Ambion, USA) before storage at -80°C.

The RNA was extracted from liver samples (5 replicates from each group) using RNeasy Mini Kit (Qiagen, USA) as per manufacturer’s protocol. The integrity of total RNA was checked on 1% agarose gel electrophoresis using 1X Tris-acetate-EDTA (TAE) buffer. The bands of 28sRNA and 18sRNA reflected the quality of extracted total RNA. The purity of total RNA (free from protein and genomic DNA contamination) was checked using nanodrop by OD 260:OD 280 values which was recorded to be >1.8. The first strand cDNA was synthesized from purified RNA using SuperscriptVILOcDNA synthesis kit (Invitrogen, USA). From each liver sample, 2.5 μg of total RNA was reverse transcribed using 4 μl of VILO reaction mixture and 20 μl of Superscriptenzyme mixture to make a final volume of 20 μl with nuclease free water in a sterile PCR tube on ice. This reaction mixture was incubated at 25°C for 10 min, later at 42°C for 60 min, after which the reaction was terminated (85°C for 5 min). The cDNA was stored at −20°C.

Real time quantitative polymerase chain reaction (qRT-PCR) analyses were performed with three replicate per sample of each gene using the STEP ONE PLUS real time PCR system (Applied Bio systems, USA), Fast SYBR Green master mix (Applied Bio systems, USA) and gene specific primer sequence for both reference and target genes (Table 3).

**Table 3:** Gene primer sequence of SOD1 and reference gene.

Gene	Primer Sequence	Amplicon size	Accession No
SOD1	Forward:5’GACCACTTTAACCCTGATGGAA	183bp	JN033790.1
	Reverse: 3’GGTCATCTTCTCCCTCATCAAC		
GAPDH	Forward: 5'ATGGGCGTGAACCACGAGAA	146bp	NM_001190390.1
	Reverse: 5'-ATGGCGTGGACAGTGGTCAT		

Glyceraldehyde phosphate dehydrogenase (GAPDH) was used as the reference gene in this study. Primers were designed using the Primer3 program [18] with an annealing temperature of 60°C and amplicon size of less than 250 bp. Primer efficiencies were checked by 10-fold serial dilution of cDNA [19] and observed to be 101.5 and 103 for reference and target gene, respectively. The PCR condition used to amplify all genes was initial denaturation at 95°C for 20 sec with 40 cycles of denaturation at 95°C for 3 sec followed by annealing and extension at 60°C for 30 sec and a melt curve of 95°C (15 sec), 60°C (1 min) and 95°C (15 sec). The specificity of each PCR product was determined by a melt curve analysis and amplicon size determination by agarose gel (0.2%). Negative controls which consisted of all components of qRT-PCR mix except cDNA were used for both the primers. The Ct (threshold cycle for target amplification) values were analyzed using 2 ^-ΔΔCt^ (normalized expression ratio) method to determine the relative level of expression of each mRNA [20]. ΔCt = Ct (target gene)–Ct (housekeeping gene) and ΔΔCt = ΔCt (target gene sample)–ΔCt (calibrator).

### Statistical analysis

The data were analysed using Statistical Package for Social Sciences (SPSS, version 20.0; Chicago, USA) by two-way ANOVA and comparison of means was tested using Duncan’s multiple range tests [21]. The analysis included 2x2 between subjects factorial design (Ca main effect, B main effect and interaction between the Ca and B (Ca x B). In case of CMI, data recorded was analysed with dietary group and time interval (h) as repeated measures and interaction between dietary group and time interval (h) as main effects. The effects were considered to be significant at P<0.05 or P<0.01.

## Results and discussion

### Low Ca decreases average daily gain, but boron supplementation restores it

The growth performance of lambs fed on the different experimental rations is presented in the Table 4. The average daily gain (ADG) was significantly (P<0.05) lower in lambs fed Ca-inadequate diet (LCa), but the same was restored and values were at par with normal Ca (NCa) control upon B-supplementation. This might be due to the ability of B to improve Ca utilisation which, in turn, might have enhanced the digestive process to facilitate higher nutrient utilisation in LCa group thus improving body weight gain on B-supplementation to low Ca diet. In contrary, earlier studies in farm animals have reported no influence of B-supplementation on body weight gain in growing steers [9], Merino withers [22], gilts [9] and barrows [23].

**Table 4:** Growth performance of lambs fed experimental diets.

Attributes	Dietary groups^†^				P-value	Factors		
	NCa	LCa	NCaB-40	LCaB-40		Ca	B	Ca*B
Initial body weight (kg)	17.8±0.71	17.8±1.02	17.6±0.52	17.9±0.48	0.986	0.818	0.927	0.783
Final body weight (kg)	28.5±0.74	27.2±0.83	28.6±0.39	28.7±0.28	0.292	0.818	0.183	0.283
Weight gain (kg)	10.6^a^±0.20	9.4^b^ ±0.27	11.1^a^ ±0.20	10.8^a^ ±0.35	<0.01	<0.05	<0.01	0.092
ADG (g)	59.1^a^±1.09	52.4^b^ ±1.52	61.5^a^ ±1.10	60.0^a^ ±1.97	<0.01	<0.05	<0.01	0.092

^†^Normal-calcium diet (NCa) and Low calcium diet (LCa) supplemented with 40 ppm boron (NCaB-40 and LCaB-40)

^ab^Means bearing different superscripts in a row differ significantly (P<0.05; P<0.01)

ADG: average daily gain; Ca: Calcium; B: Boron;

* Interaction

### Low dietary Ca lowers total antioxidant activity, relative mRNA abundance of SOD1 and humoral and cellular immune responses, but Boron supplementation restores/ameliorates it

**Fig 1:**
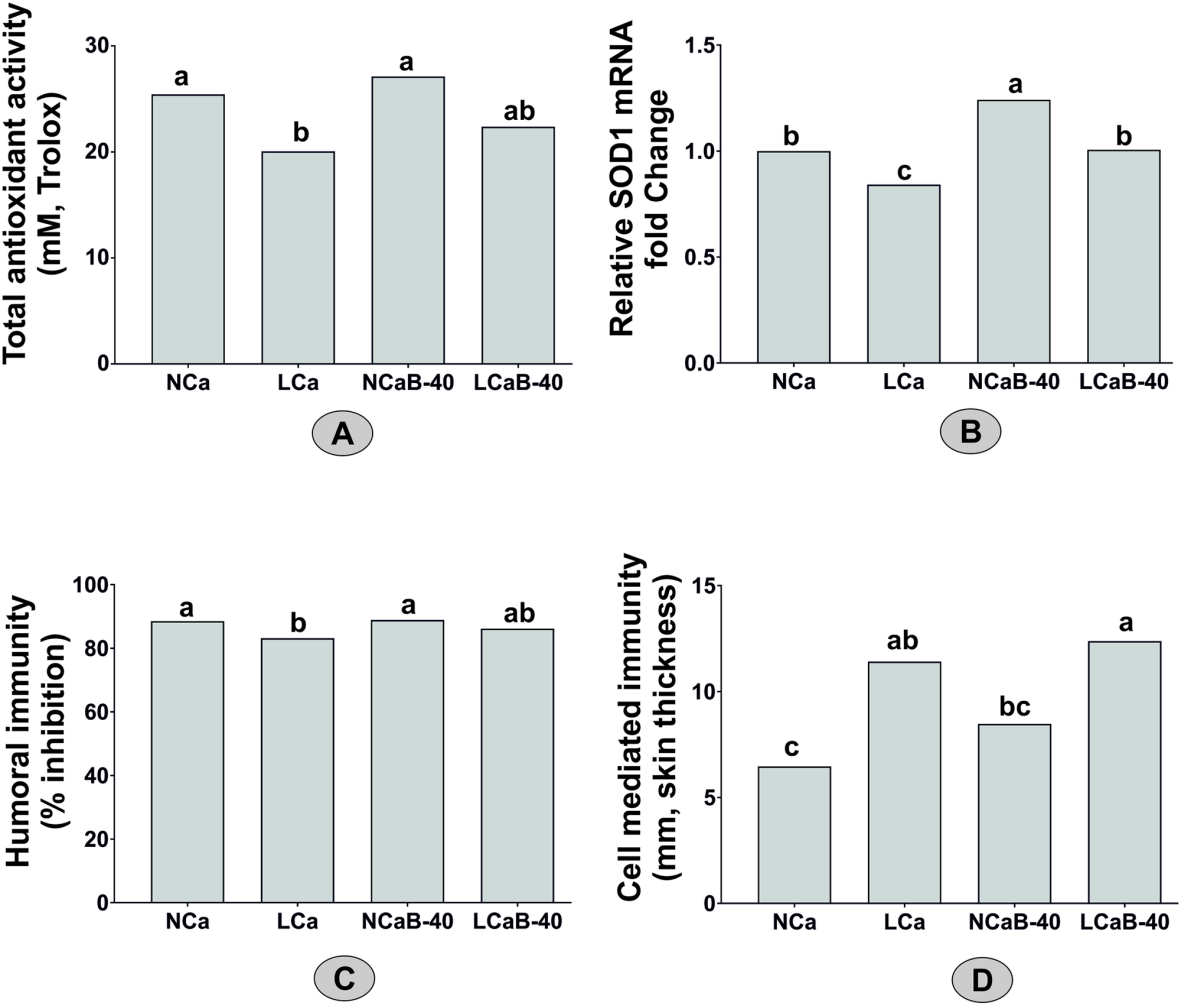
Influence of boron supplementation on total antioxidant activity, relative mRNA abundance of SOD1, humoral and cellular immune responses of lambs fed diets with or without adequate level of calcium. Note: lowered total antioxidant capacity(A), relative mRNA abundance of SOD1(B) and humoral immune responses (C) on low Ca diets are restored and cellular immune response (D) is ameliorated by Boron supplementation.

Influence of boron supplementation on total antioxidant capacity, relative mRNA abundance of SOD1, humoral and cellular immune responses of lambs fed diets with or without adequate level of calcium are summarized in Fig 1. Serum SOD levels are given in Table 5.

**Table 5:** Serum SOD activity in ram lambs fed on experimental rations.

Attributes	Dietary groups^†^				P-value	Factors		
	NCa	LCa	NCaB-40	LCaB-40		Ca	B	Ca*B
SOD (Units/mg Hb)	738±34.18	671±49.40	777±31.64	718±21.32	0.262	0.098	0.249	0.915

^†^Normal-calcium diet (NCa) and Low calcium diet (LCa) supplemented with 40 ppm boron (NCaB-40 and LCaB-40)

Ca: Calcium; B: Boron; SOD: Superoxide dismutase; Hb: Haemoglobin;

* Interaction

The TAA was significantly (P<0.05) lower in lambs fed LCa diet and became comparable (P>0.05) to that of the control values upon 40 ppm B-supplementation (Table 5). Total antioxidant activity is indicative of the counteracting effects of the antioxidants against oxidative stress-induced damage in cells.

Humoral immune response against the PPR vaccine was lower (P<0.05) in LCa diet fed group and was restored to become comparable to the control values on B-supplementation (LCaB-40). Upon antigen encounter, cytokines released from T-cell help the B-cell to multiply and mature into antibody producing plasma cells, which are released into blood to form antigen-antibody complexes. Previous studies also indicated that dietary B (5 ppm) was beneficial in subsiding (P<0.05) inflammatory response to an intra-dermal injection of phytohemagglutinin in gilts [8]. In the same line, serum concentrations of plasma cytokine in growing steers fed dietary B (5 ppm) increased after inoculation with bovine herpesvirustype-1 [9].

The mRNA expression of SOD1 gene in liver was significantly (P<0.05) lower in lambs fed LCa diet, and B-supplementation to both LCa diet as well as control diet enhanced (P<0.05) its expression (Fig 1). However, the same could not be translated in terms of a higher SOD activity in the serum (Table 5). These findings suggest an upregulatory effect of B supplementation on hepatic mRNA expression levels of SOD1 under the conditions of abiotic stress induced by dietary Ca restriction. Similar observation has been recorded in rats fed purified diets supplemented with B [10]. To the best of our knowledge, this is the first report suggesting the influence of dietary B on expression of SOD1 mRNA in liver tissue of sheep fed diets adequate or deficit in Ca. Improved antioxidant and immune status coupled with increased hepatic mRNA expression of SOD1 with B supplementation to low Ca diets suggest a plausible role of B in ameliorating stress and improving health in animals. However, further studies are required to fine-tune its optimum dietary levels for various functional roles, and to establish the physiological role of B in improving immunity and health in farm animals. Similarly, beneficial action of dietary B in enhancing the activity of SOD in liver, erythrocytes and kidney of rats have been reported [10, 24]. Superoxide dismutase 1 is a Cu and Zn dependent antioxidant enzyme is known to scavenge the reactive oxygen species, serves as a key antioxidant in protecting the cell from superoxide toxicity thereby help relieving oxidative stress. Thus, it can be deduced from present study that dietary B improves antioxidant defence mechanism under stress conditions.

The DTH response, representing the cell-mediated immune response, was significantly (P<0.05) higher in lambs fed LCa diet without and with B-supplementation (LCaB-40) (Fig 1). No significant interaction was observed between the levels of either Ca or B in diets with the period of measuring the skin thickness (Table 6). Increase in skin thickness (mm) after mitogen injection considered as an index of CMI in the present study could be due to the infiltration of cytokines, interleukins and macrophages. Previous studies indicated that dietary B-supplementation (5 ppm) increased the serum concentrations of tumour necrosis factor-α (TNF-α) (produced by activated macrophages) and cortisol after intradermal injection of 25 μg of LPS/kg body weight in pigs [7]. The overall improved immune response with B-supplemented diets observed in the present study clearly suggests that B potentiates the immune responsiveness under Ca deficit conditions and demonstrates an immunomodulatory role.

**Table 6:** Cell mediated immunity response of ram lambs fed on experimental rations.

Attributes	Dietary groups†				Mean	Factors	
	NCa	LCa	NCaB-40	LCaB-40		Within subjects	Between subjects
Cell mediated immune response (skin thickness in mm)							
12h	10.4±0.87	15.0±0.84	13.4±1.64	15.6±3.59	13.6^a^±1.07	H <0.01	Group <0.05
24h	6.01±0.89	13.1±1.60	8.63±1.80	14.7±2.24	10.6^b^ ±0.79	H*Ca 0.08	Ca <0.01
36h	5.28±0.60	10.8±1.22	6.44±0.81	10.8±1.72	8.34^b^^c^ ±0.57	H*B 0.79	B 0.23
48h	4.18±0.15	6.74±0.86	5.47±0.42	8.40±1.53	6.20^c^ ±0.45	H*Ca*B 0.85	Ca*B 0.65

^†^Normal-calcium diet (NCa) and Low calcium diet (LCa) supplemented with 40 ppm boron (NCaB-40 and LCaB-40);

* Interaction; H: Hour; Ca: Calcium; B: Boron

^abc^ Means bearing different superscripts in a column differ significantly: P<0.01; P<0.05

### Dietary Ca restriction induces tissue histological alterations, but Boron supplementation ameliorates it

The B-supplementation to normal Ca diets did not induce any histopathological changes in spleen and it appeared to have retained its normal architecture in all the dietary groups. Supplementation of B (NCaB) to control ration did not reveal any tissue abnormality in any of the visceral organs (liver, kidney, spleen) of lambs. Previous reports indicated that B-supplementation (50 ppm) did not alter the histological features of liver, kidney, spleen and thymus in rats [25]. Similarly, B-supplementation at 100 ppm has been reported to improve growth of immune organs like spleen, thymus and bursa in broilers [26]. Thus, it clearly suggests that a dosage of dietary boron at 40 ppm may not induce pathological changes in liver, kidney and spleen tissues.

**Fig 2:**
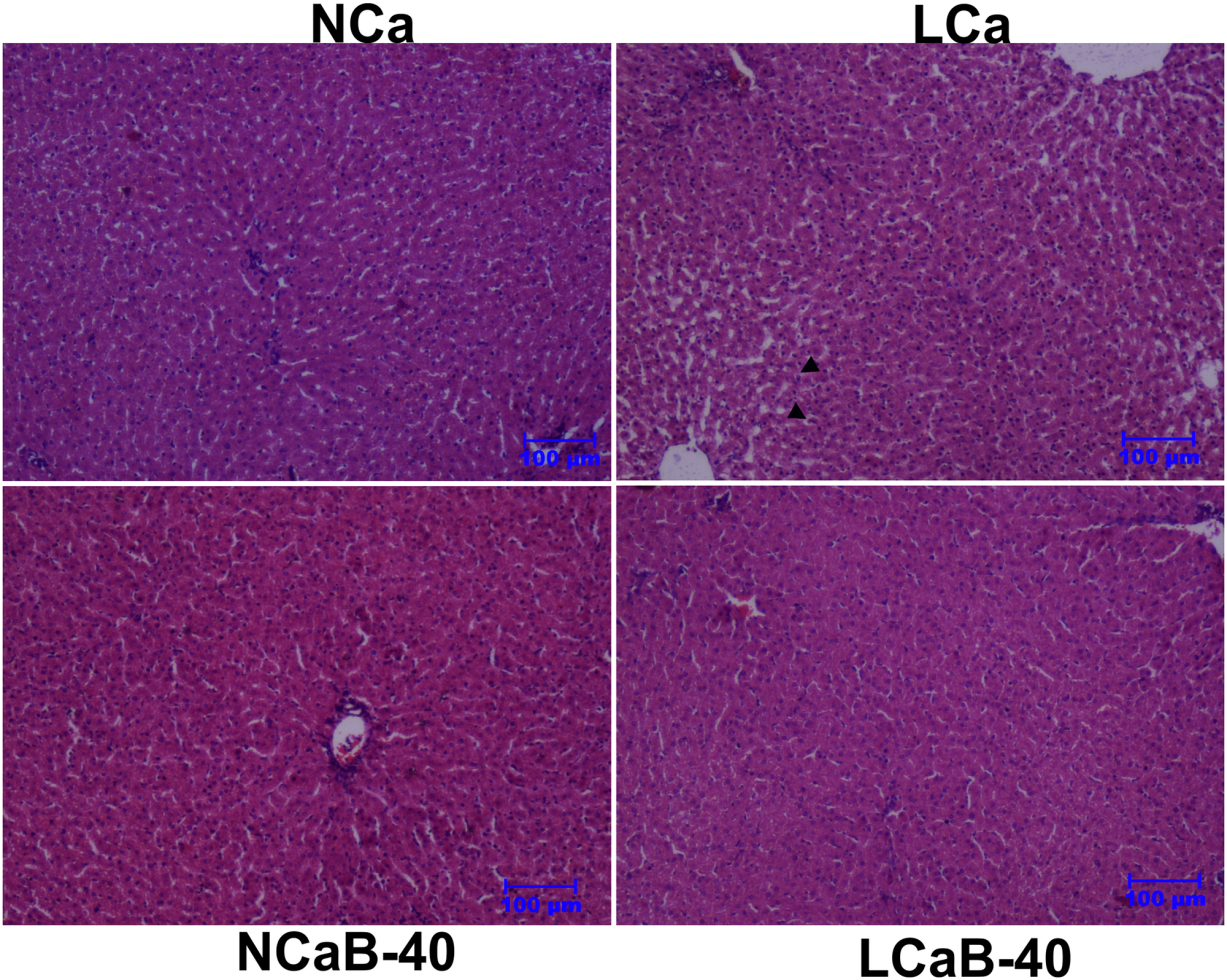
Pictomicrograph showing liver tissue in ram lambs fed experimental rations. Haematoxylin and Eosin staining, scale bar = 100μm. Ca deficiency induced histological alterations are restored/ameliorated by Boron. Arrows in the LC group indicate vacuole degeneration and nuclear condensation.

**Fig 3:**
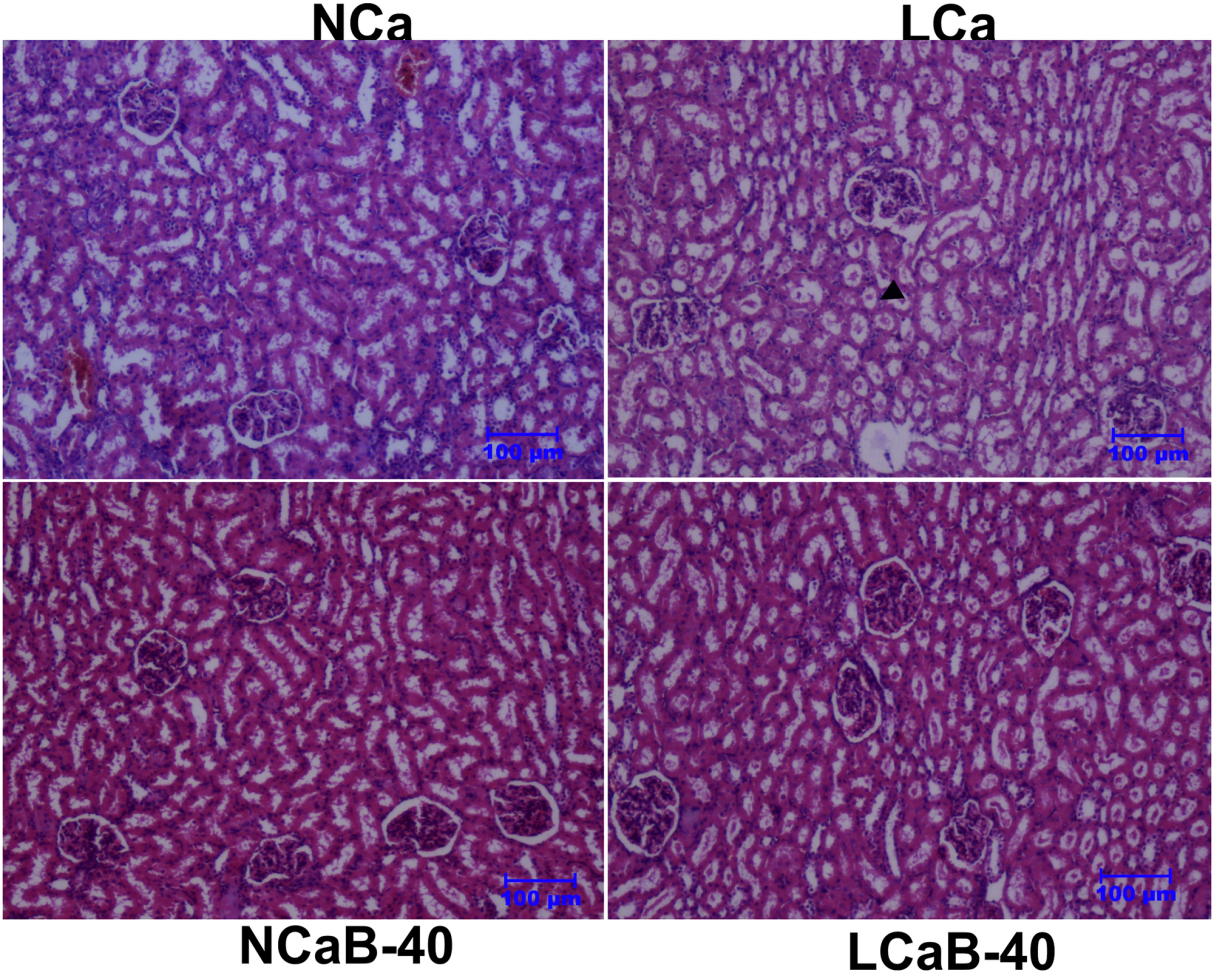
Pictomicrograph showing kidney tissue in ram lambs fed experimental rations. Haematoxylin and Eosin staining, scale bar = 100μm. Ca deficiency induced histological alterations are restored/ameliorated by Boron. Arrows in the LC group indicate protein casts.

Examination of the liver tissue revealed mild vacuolar degeneration of hepatocytes around the central vein and condensation of nucleus with distortion in architecture of hepatic cords in lambs fed LCa diet (Fig 2). In the LCa group, certain degenerative changes were observed in hepatocytes with loss of cellular architecture, distortion of hepatic cords and dilatation of the central vein. This might be due to the fact that reversible cell injury occurs when cytosolic Ca^+2^ ions increased with enhanced release of Ca^+2^ from mitochondria and endoplasmic reticulum under Ca deficit conditions. The B-supplementation to Ca deficient diet (LCa-40) reduced the severity of lesions both in liver and kidney which was evident by the histological changes resembling to that of control group (NCa). The ameliorative effect of B in the present study is supported by the report that physiological level of boric acid prevent the release of Ca^+2^ from endoplasmic reticulum and helps in reducing the risk of cancer [27]. Kidney tissue showed moderate accumulation of proteinaceous casts in the lumen of tubules with mild damage to the glomerulus inside the Bowman's capsule in LCa group (Fig 3). Similarly, kidney tissue in lambs of LCa group showed moderate accumulation of proteinaceous casts in the lumen of the tubules with mild damage to the glomerulus. This could be due to altered P metabolism (hypophosphataemia) in order to stabilize the Ca: P ratio under Ca deficit conditions. Boron supplemented at 40 and 80 mg/L in drinking water for 90 days could not induce pathological lesions in kidney tissue of ostrich, however, higher doses of 320 and 640 mg/L induced tubular degeneration [28]. Further, dietary supplementation of 20 ppm B in rats ameliorated the pathological effects in kidney induced by malathion toxicity [29].

**Fig 4:**
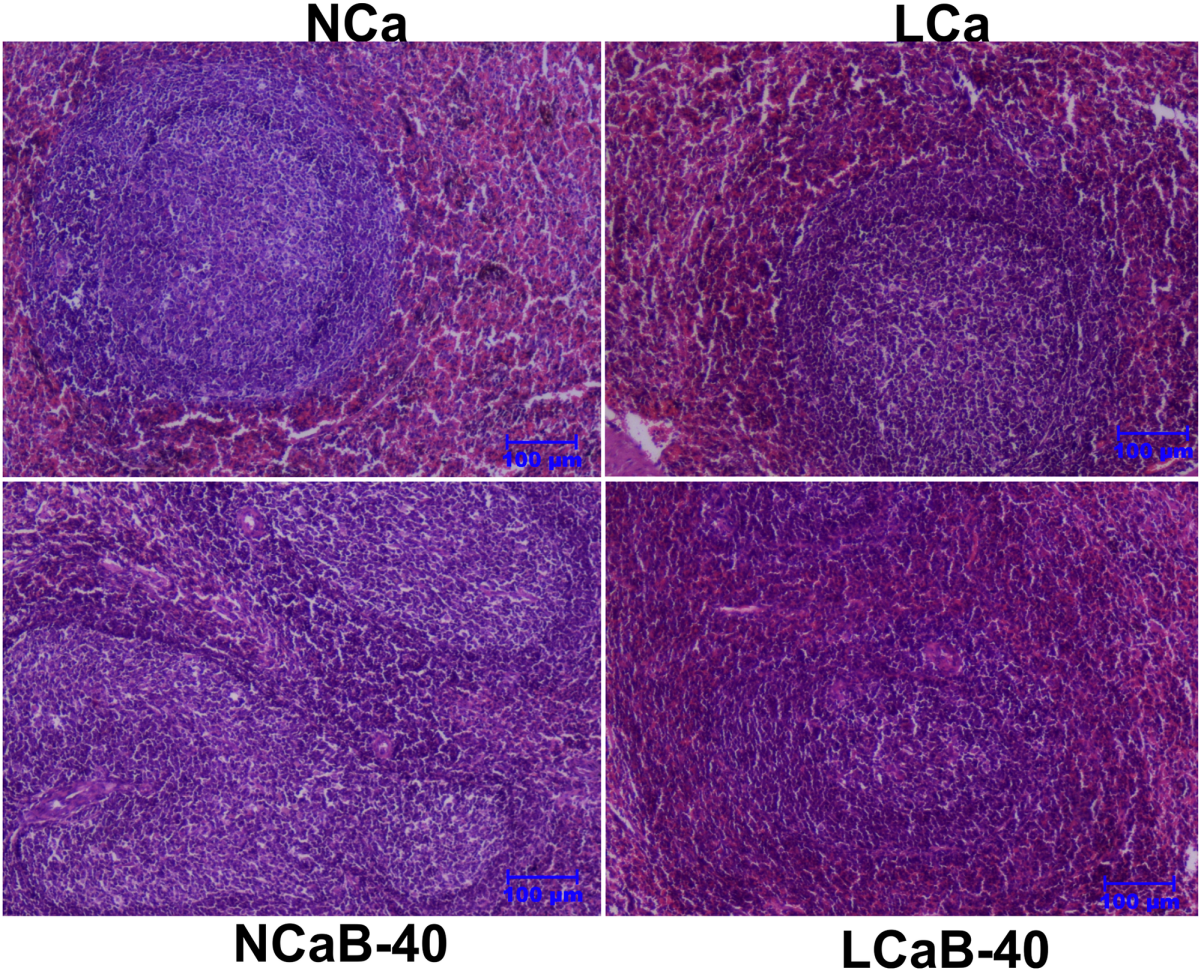
Pictomicrograph showing spleen tissue in ram lambs fed experimental rations. Haematoxylin and Eosin staining, scale bar = 100μm. Ca deficiency induced histological alterations are restored/ameliorated by Boron.

The spleen did not reveal any observable changes when compared to control group (Fig 4). In the same line, B-supplementation at 40 ppm reportedly improved the spleen tissue structure, while at 80 ppm B damaged the splenic structure in rats [30]. This supports the health promoting effect of boron at physiological level [31].

## Conclusions

This study concluded that feeding diets low in Ca results in lowered growth rate, antioxidant status, immune response along with histological alterations in liver and kidney. However, supplementation of B (40 ppm) in such Ca-deficit diets restores some of these immune-biochemical changes and or has an ameliorating effect on tissue histoarchitecture.

## Supporting information

S1 Data Readings recorded during the experiment pertaining to the results presented in this manuscript. (XLSX)
